# A Rare Case of Plasma Cell Gingivitis with Cheilitis

**DOI:** 10.1155/2019/2939126

**Published:** 2019-12-16

**Authors:** Yashodeep Chauhan, Shaleen Khetarpal, Madhu Singh Ratre, Manish Varma

**Affiliations:** Department of Periodontology, Govt. College of Dentistry, Indore, M.P., India

## Abstract

**Background:**

Plasma cell gingivitis (PCG) is a rare condition of the gingiva, characterized histopathologically by infiltration of plasma cells in connective tissue. Hypersensitivity reaction due to antigen is considered as primary etiological factor.

**Case Presentation:**

The present case is of an 18-year-old male patient suffering from gingival enlargement along with cheilitis. Histopathological and immunohistochemistry of tissue revealed lesion as plasma cell gingivitis. After gingivectomy, the follow up of the patient was done for 8 months. Gradual reduction of lip swelling was observed after gingivectomy during subsequent visits.

**Conclusion:**

Early diagnosis is essential as plasma cell gingivitis has similar pathologic changes seen clinically as in leukemia, multiple myeloma, discoid lupus erythematosus, atrophic lichen planus, desquamative gingivitis, or cicatricial pemphigoid which must be differentiated through hematologic examination.

## 1. Introduction

Plasma cell gingivitis (PCG) is a rare benign inflammatory condition of the gingiva. Various synonyms of plasma cell gingivitis are atypical gingivostomatitis, idiopathic gingivostomatitis, allergic gingivostomatitis, and plasma cell gingivostomatitis [1]. The lesion was first described in the year 1952 when Zoon referred to the term as “plasma-cell infiltrate.” These conditions have also been reported on the lips, tongue, vulva, conjunctiva, nasal aperture, larynx, and epiglottis [2]. PCG is caused by allergen which induces hypersensitivity reaction. Various allergens documented are chewing gums, certain components of toothpastes, cinnamon, mint, red pepper, and khat leaves [3]. However, PCG has been classified into three categories, based on the etiology as follows: PCG due to allergens, PCG due to neoplastic origin, and PCG due to unknown cause [4]. Clinically, PCG is characterized by sharply demarcated erythematous and edematous gingiva often extending to the mucogingival junction. Moreover, the gingiva appears red, friable, and bleeds easily on provocation [1]. PCG mimics lesions associated with discoid lupus, lichen planus, cicatricial pemphigoid, leukemia, and myeloma; thus, an early diagnosis in such cases is vital to the patient's interest [1]. The case presented here is a PCG associated with cheilitis in an 18-year-old male patient.

## 2. Case Presentation

**Figure 1:**
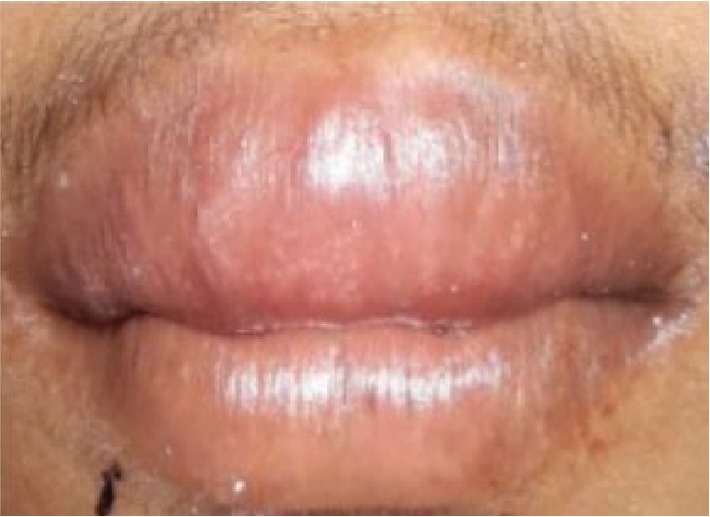
Cheilitis in the lips.

**Figure 2:**
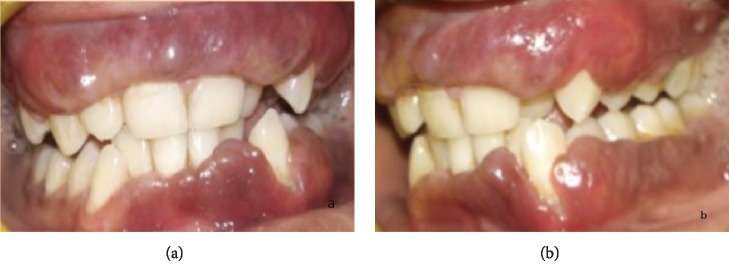
Gingival enlargement.

Patient, aged 18 years old, male, reported to the Department of Periodontics with the chief complaint of unaesthetic swelling of the gums in the maxillary and mandibular regions of the teeth since 4 to 5 years. Extraoral examination revealed cheilitis (Figure 1). Intraoral examination revealed generalized severe gingival enlargement covering up to the middle third of the clinical crowns. Gingiva was red, oedematous, and friable, with the absence of stippling, and easily bleeds on provocation. Gingival enlargement extends from teeth 14 to 27 in the maxilla and 37 to 43 in the mandible (Figure 2). Minimal local deposits were found in the mouth. There was no loss of attachment; however, generalized pseudo pockets ranged from 6 mm to 8 mm were recorded. The medical, dental, and personal history of the patient was noncontributory. Investigative hematologic examination did not reveal any significant findings. There was a negative Nikolsky's sign with no cutaneous lesion. Excisional biopsy was done to rule out PCG.

**Figure 3:**
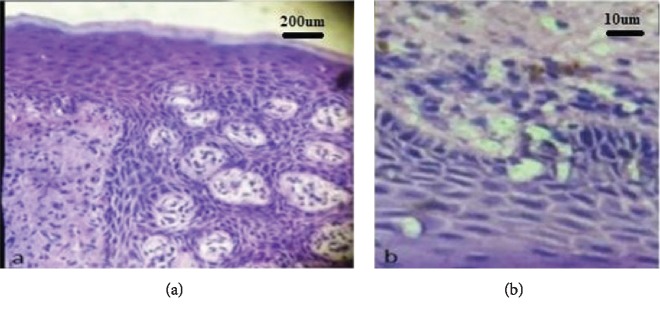
Histopathology: (a) 20x magnification and (b) 100x magnification.

**Figure 4:**
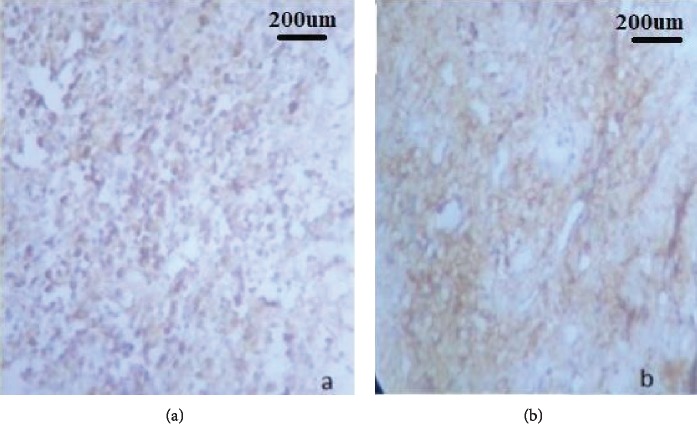
Immunohistochemistry showing positive reactivity for (a) kappa chain and (b) lambda chain.

Histopathological examination revealed parakeratinized stratified squamous epithelium of varying thickness with regions of thinned-out epithelium and sharp rete ridges (Figure 3). Connective tissue was composed of dense diffuse chronic inflammatory cell infiltrate predominantly of plasma cells, lymphocytes, collagen fibers, and endothelium lined blood vessels. Above features were indicative of plasma cell gingivitis. The immunohistochemistry study further confirmed the lesion with the aid of kappa and lambda light chain reactivity (Figure 4). No relevant radio graphical finding was present.

**Figure 5:**
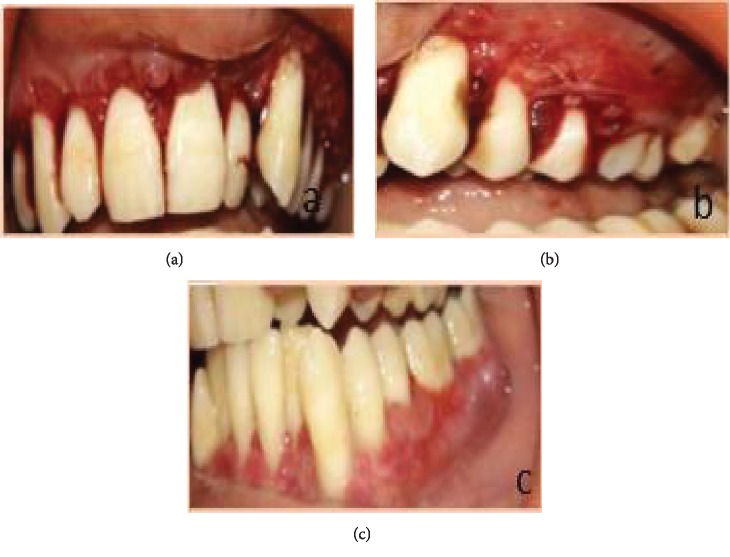
Immediate postoperative view: (a, b) maxilla and (c) mandible.

**Figure 6:**
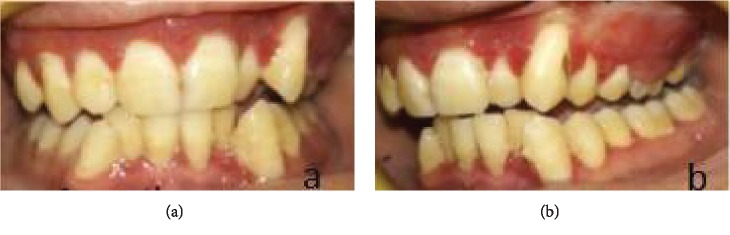
Follow-up at 1 month.

**Figure 7:**
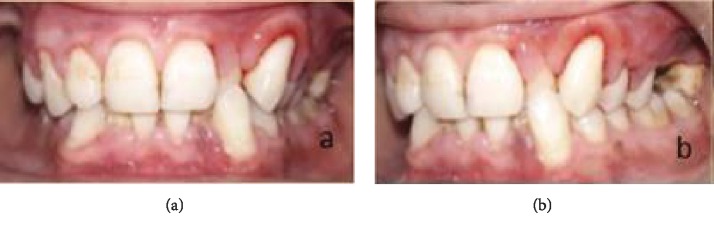
Follow-up at 3 months.

**Figure 8:**
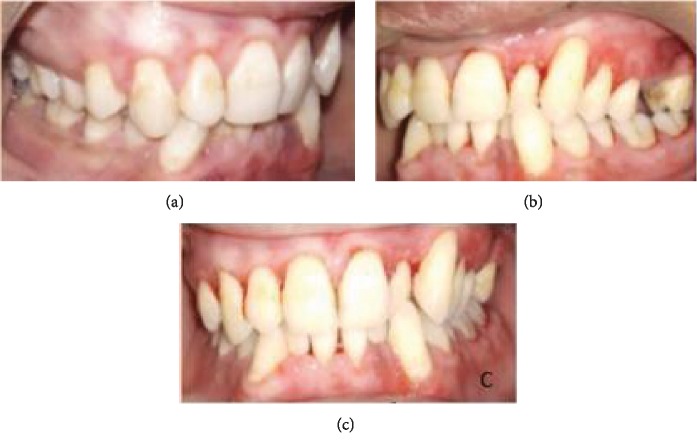
Follow-up at 8 months.

**Figure 9:**
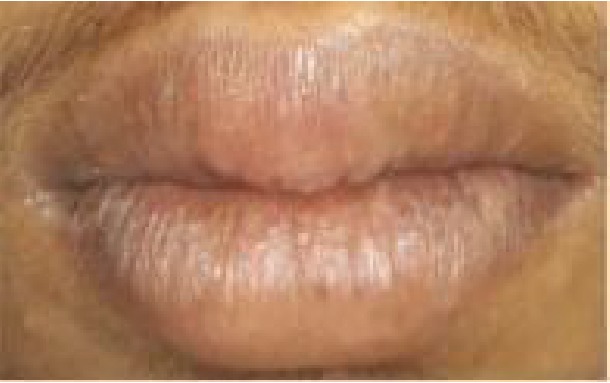
Reduction in lip swelling.

After diagnosis, treatment was planned as phase I therapy followed by external bevel gingivectomy procedure under local anesthesia. The procedure was explained to the patient, and consent was taken. Postsurgical healing was satisfactory, and no recurrence was observed at consecutive follow-up visits. Follow-up of the patient was done at 1 week, 2 weeks, 3 weeks, 1 month, 3 months, 6 months, and 8 months (Figures 5–8). Gradual decrease in lip swelling was observed (Figure 9).

## 3. Discussion

PCG is a rare inflammatory condition, characterized by diffuse and massive infiltration of the plasma cells into the connective tissue [5]. Kerr and Kenneth in 1981 reported gingival enlargement in gum chewers, which disappeared following the discontinuation of the chewing habit [6]. Gargiulo et al. classified PCG as an immunological reaction to allergens, neoplasia, or of unknown origin [4]. Antigenic identification is necessary for proper diagnosis of the condition along with clinical, histopathological, and hematological screening [5]. However, in the present case, identification of antigen was unattainable; therefore, it was classified as the third variant of PCG. PCG resembles histologically as multiple myeloma and plasmacytoma, clinically as acute leukemia. Clinically, PCG is characterized as oedematous swelling with diffuse erythema clearly demarcated from mucogingival junction [1]. In the present cases, gingival enlargement was confined to both maxillary and mandibular teeth which was fiery red in color and obstinate to oral prophylaxis. These findings are consistent with earlier cases as documented by Joshi and Sukla [1]. Although in contrast to the present case, Arduino et al. found a decrease in gingival enlargement after phase I therapy [7]. In contrast to the case reported by Kumar et al., in present case, loss of attachment and severe bone loss were not appreciated [8]. In contrast to the present case, Makkar et al. reported a case of PCG in a 17-year-old female with generalized aggressive periodontitis [9]. As presented in a most recent case series by Prasanna et al., a similar case of PCG associated with cheilitis, they also reported subsiding of cheilitis after treatment as in our case [5]. Gingivitis, cheilitis, and glossitis have been described as a triad for plasma cell gingivostomatitis [6]. In the present case, removal of the gingival lesion by gingivectomy resulted in the reduction of lip swelling suggesting that the combined lesion of the lip and gingiva could be due to contact dermatitis as labial mucosa was in close contact of the gingiva as reported by Abhishek and Rashmi [10]. Histopathologically, it is crucial to differentiate PCG from various plasma cell tumors. The gingival contour and texture were stable after 8 months of gingivectomy. Gradual decrease in lip swelling was observed during consecutive follow-up visits. Diagnosis by clinical exclusion, haematological, histopathological examination, and immunohistochemistry helps to arrive at a diagnosis of PCG and inflammatory cheilitis.

## 4. Conclusion

As PCG mimics various other fatal conditions such as leukemia and multiple myeloma, early diagnosis and prompt treatment of the lesion are necessary. Therefore, a careful case history taken along with hematological, histopathological, and immunohistochemical examination is necessary so as to exclude other lesions and come to a proper diagnosis.
